# Treatment of Pyorrhœa by the General Practitioner

**Published:** 1916-08

**Authors:** O. S. Clappison

**Affiliations:** Hamilton, Ont.


### TREATMENT OF PYORRHŒA BY THE GENERAL PRACTITIONER.

O. S. CLAPPISON, D.D.S., L.D.S., HAMILTON, ONT.

We know that the beginning of pyorrhœa is a gingivitis, the chief feature of *which is some sort of traumatism of the circular ligament.* Constitutionally a lowered *resistance seems necessary to the development of the disease,* and locally the normal attachment is loosened, the soft tissues altered into granulating tissue with more or less pus formation.

It is the opinion of many prominent dentists today that there are no systemic reasons for the cause of pyorrhœa other than those which may predispose to any disease. For instance, Hutchinson says:

"If diabetes, syphilis, tuberculosis or other systemic disorders are coincident with pyorrhœa, the pyorrhœa antidates the constitutional disorder, and has been accentuated but not caused by such disorder, and if the mouth has been under proper prophylactic treatment I believe there would have been no pyorrhœa."

Among the local causes are:

1. Deep interlocking cusps in bridge work.
2. Poorly constructed bridge work.
3. Partial dentures.

4. Ligatures, clamps, wedges, etc.
5. Malocclusion of natural cusps, fillings or crowns.
6. Faulty contact points.
7. Any mechanical irritation lodged under the free margin of the gums.
8. Ragged enamel at the junction of crown and root of teeth.
9. Formation of calculus.
10. Uncleanliness.
11. Bacterial invasion and parasites.

Dr. Hartzell says: "Granted that the gingival margins play an important part in this disease. If the gingival inflammations continue, what is the explanation? Is it because the tissues of the gingivæ are less well protected? Is it because the blood supply is less generous than other areas in the oral cavity? Or is it because irritating agents accumulate on the tooth's surface and constantly flow under the gingivæ? The *bacterial* masses on the tooth's surface furnish *the chief irritant,* and the imperfectly protected gum margins permit the irritant to enter the tissues. The blood supply being received from the end capillaries, the return flow is checked by swelling, thus creating a favorable field for bacterial invasion. Every dentist has noticed the low grade gingivitis on the lingual surface of the lower incisors, which because of their position are particularly subject to the accumulation of salivary calculus. The tissues recede, the process is broken down around these teeth, accompanied by comparatively slight inflammatory disturbance, and often no evidence of pus. It will also be noticed that gingival inflammation does not occur on the gum margins of patients who have consistently practiced oral prophylaxis on the tooth surfaces, so that the tooth surfaces are free from bacterial and mechanical irritants." We can, therefore, infer from these conclusions that *"clean tooth surfaces and hard gums prevent gingival inflammations."*

If a disclosing stain is applied to teeth, the enamel of which to the casual observer appears to be clean, in many cases a dark gingival discoloration results, showing the presence of muco-plaque material, and although the patient may insist that the teeth are brushed frequently and effectively, the fact remains that they do not properly manipulate the tooth brush so as to maintain that polished condition of the teeth so necessary in the prevention of this gingival irritation.

For a differential diagnosis we have to consider:

1. *Loosening of the teeth* from faulty articulation. A large percentage of the pyorrhœa we are called upon to treat arises as a result of this condition.

2. Traumatism. Note all ill-fitting bands, fulty contacts, etc.

3. Senile atrophy.

4. Precocious atrophy.

5. Pus exuding from the gingivæ may be due to—
   (a) Calculus.
   (b) May be due to an alveolar abscess draining at the gingivæ.
   (c) To heavy metals, namely, mercury, lead, etc.

### PROGNOSIS.

Thanks to the efforts of such men as Gysi, Cummer, Chayes and others, the loss of a few teeth does not occasion the anxiety formerly felt.

Modern prosthesis is able to supply efficient artificial substitutes, and in very advanced cases the resorting to prosthesis seems to be the solution of the difficulty and more conducive to the patient's general health. In the majority of cases, however, in which the disease has been correctly diagnosed in its early stages, its etiology plainly recognized, the progress should be favorable, bearing in mind that the X-ray is a valuable adjunct in many cases. The most apparent uses of the radiograph are to show:

1. The amount of bone destruction and absorption around the roots of the affected teeth.
2. The presence of undiscovered blind abscesses which may be discharging at the gingivæ.
3. Absorbed and eroded roots.
4. Excementosis.

TREATMENT.

Let us become reconciled to the fact that there is no royal road leading to the cure of this disease, but he who wishes to successfully treat this condition might better place his trust in skillful operating rather than in a hypodermic syringe, tablets, or other remedies.

*The only "sure cure" for pyorrhœa is "never to let it begin."* Too many nostrums are to be had, and are in use today, and, sad to relate, too many, the composition of which most of the dentists who use them are absolutely ignorant. In many instances more beneficial and lasting results could be obtained by the judicious use of Epsom salts. But let us get away from this word *"cure,"* and stop creating a false impression on our patients. Let us be honest in telling them that we do *not cure their pyorrhœa, but that we do offer them relief* from the acute symptoms, and, where possible, put their teeth into a comfortable condition, educating them in proper prophylaxis of the mouth, and have them return at stated intervals for similar treatment. It is obvious that by asking them to return for treatments we admit that the condition has not been permanently cured.

One of the many questions asked the dentist when a patient is informed of the presence of pyorrhœa is, "Can it be cured?" "Will it stay cured?" by the term *cure* in this connection let us take it that what is meant is—

1. The total disappearance of pus.
2. The filling in and obliteration of pockets.

3. The return of the gums to a normal pink color, becoming hard and firm.

4. A tightening of the loose teeth affected.

Your reply in such cases would undoubtedly depend upon the stage to which the disease had progressed, and it may not be out of place just here to suggest that the time to treat this condition is at the first sign of gingival inflammation, and not when the teeth are loose and pus is exuding from the pockets. When the treatment is instituted the patient must understand that where the gums have receded and the necks are exposed, calculus will readily collect which can not easily be removed by the patient, and we must impress upon them the necessity of frequent visits in order that the tissues may be kept free from this irritant.

*The first requisite in the treatment of pyorrhœa is tooth cleanliness.* Each and every tooth must first be freed of all deposits on its exposed surfaces. Roughened surfaces made smooth, overhanging margins of fillings cut down by the use of Smith's files, or by stones, discs and strips; exposed necks of teeth polished with orangewood sticks, tape and powder, and, where possible, ill-fitting crown, dentures, etc., eliminated. It would be advisable at this stage to note all faulty contacts, and, where possible, cut a cavity into the tooth or old filling and pack with gutta percha, so that at some future sitting the contact may be restored and a filling with a well-formed marginal ridge inserted in order that the interproximal tissues may remain healthy. This should be done for every patient, whether or not we treat the pyorrhœa.

By polishing exposed surfaces, removing overhanging fillings, etc., before scaling the roots we accomplish several purposes.

1. Gingival inflammation and hemorrhage are lessened.
2. Insoluble powder is not forced into the pockets.
3. Large areas of infection are not opened.

4. If the tissues are very sensitive, the application of the iodo glycerole preparation, or other medicinal remedy, will render the tissues less sensitive and more amenable to treatment.

As the writer has stated before, it is a lamentable fact that so few patients possess sufficient digital dexterity to properly manipulate a tooth brush, and no time spent by the dentist is productive of greater results than that time devoted to demonstrating to the patient the proper technique of brushing the teeth, and having the patient practice this technique until the results obtained are such as will be productive of a more hygienic mouth, and until the operator is convinced that the patient is securing the best possible results. At each visit the condition is noted, and attention drawn to surfaces that have not been properly polished, or a *disclosing solution* applied and the patient is shown the condition resulting from neglect to reach every possible angle. It may be necessary to repeat this performance many times, but it must be evident that to a large extent success or failure in this treatment hinges on the ability of the operator to induce the patient to maintain this ideal condition. On the other hand, however, many patients are beyond the persuasive powers of the most convincing practitioner, and it matters not how careful the explanation, or how laborious the demonstration, they either reach home too late at night, rise too early in the morning, or do not go home for lunch. This is the type of patient who goes to Dr. Brown six months after and tells him that, although you treated the pyorrhœa for him, it is worse now than it ever was.

INSTRUMENTATIION.

While the writer knows that the accepted technique of the leading dentists treating this condition is to select one tooth at a time and finish the instrumentation before proceeding to the next, he believes that where the hemorrhage is excessive the better mode of procedure for one not skilled in the instrumentation is at the first sitting for this purpose to remove the larger particles of calculus that lodge just under the gingival margins, and after covering all silicate fillings and porcelain inlays with wax, to use some iodine preparation, such as iodo-glycerole.

As this may not be familiar to all, I will give the formula:

℞—Iodo-glycerole:

| | |
|---|---|
| Zinc-iodide ....... | 15 parts or grams. |
| Iodine (crystals) .. | 25 parts or grams. |
| Glycerine ........ | 50 parts or grams. |
| Water ........... | 10 parts or grams. |

By slightly modifying this we obtain a disclosing solution:

| | |
|---|---|
| ℞—Zinc Iodide ................. | grs. 15 |
| Iodine (crystals) ............ | grs. 50 |
| Glycerine .................... | drs. 4 |
| Water ...................... | drs. 4 |

Put in a glass stoppered bottle.

Sig.—Paint teeth and rinse mouth immediately.

A small quill toothpick, mounted on an orangewood stick, will be found to be most serviceable for applying the iodo-glycerole. At the following sitting the hemorrhage will be found to be much less severe, and in many cases the tissues less sensitive. Where the roots are found to be hypersensitive, use silver nitrate forty per cent. to a saturated solution for the posteriors, and ten per cent. for the anteriors, and apply frequently where there is no decay. This can be applied with an orangewood stick, care, of course, being taken that the solution does not come in contact with the soft tissues.

The writer believes that he has had some success with Dr. Head's tartar solvent applied every few days where the calculus is particularly tenacious.

Every member present has seen in his every-day practice case after case where, because of early extraction of the permanent molars, the articulation has been upset, allowing the stress, which should have been taken up by the molars, to be thrown on the anterior teeth. The lower anterior teeth come into closer contact with the uppers, and the constant tap, tap, causes them to become loose and often protruded and separated. Or again, one or two teeth may be receiving more stress than the remainder, and will defy all efforts to treat until by grinding or opening the bite this abnormal pressure is relieved. It is in these anteriors that a splint is most advisable, permitting, as it does, the use of Roach, Gilmore or other attachments. The splint favored by the essayist is made by shaping the cutting edge of the incisors as for a bridge, grinding the lingual to allow for a thickness of gold, drill holes into the lingual surface straddling the pulp, or if devitalizeed, enlarging the root canal, take an impression of the lingual surface in Kerr's Compound, and make an amalgam model. Swage a gold backing in model, place in tooth, burnish to place, push a threaded post through the backing, remove and solder. Where one does not possess a Chayes parallelometer, leave the pins protruding as far as possible from backing to remain as an aid in paralleling the posts. When all the backings are in place they may either be waxed and cast or soldered as may seem fit, care being taken to leave sufficient room between the gold and the gingival margin to permit maintaining a polished condition of the neck of the tooth. By attaching Roach ball or Gilmore bar and using the compensating wires, as suggested by Dr. Cummer, a few remaining teeth may be made to render years of service, and the patient supplied with an artificial substitute giving the maximum of comfort. As a temporary splint 26-gauge gold ligature wire, with 30-gauge between the teeth, or waxed silk answers for this purpose.

The very fact that teeth have to be splinted in order that they may be saved makes them a source of frequent infection, and they will need frequent attention from the dentist to keep them in proper shape.

### INSTRUMENTS.

It is apparent that the essayist could not give a description of the technical procedure of scaling teeth, nor could any one expert form a set of scales and by placing them in the hands of every dentist expect them to become competent in their use. Each and every practitioner should select the instruments with which he can thoroughly scale the tooth surfaces without injuring the soft tissues. There are many scalers to be had, and from those secured by the essayist he has selected a few suited to his needs, some of which may be suitable for others.

For the heavier work of removing salivary calculus, or exposed particles of serumal calculus, heavy scalers are needed, together with stones, etc. For the lower incisors the writer has found a pair of Gordon White trimmers indispensable. Smith's files, Nos. 1, 2, 3, 4, 5, 6, 15, 16, will reach surfaces very difficult of access, and MacDonough's scalers Nos. 12 and 13 are excellent for the exposed roots of the molars; also any surface where a spoon-shaped instrument with both a push and pull motion is needed. An old straight, thin scaler, filed to a chisel edge, used as a push instrument, often gives quick results used between the lower incisors, where the interproximal tissues have receded sufficiently.

One of the most essential requirements in an instrument for a general practitioner is the ease with which it may be kept uniformly sharp. The writer has recently been using a set of planes, designed by Dr. Hartzell, in which the cutting bit is centered with the long axis of the instrument, and are of the plane type, which will not cut unless the plane head or shank is in contact with the surface operated on. The sharpener which goes with the set is very efficient and quick, and it is absolutely impossible to do any of this work unless the instruments are all very sharp, as it is impossible to recognize the differences in the texture of tissue with a dull instrument; hence the need of some device that will permit of uniform sharpening. This device may be secured separately, and is useful in sharpening scalers, chisels, etc.

With the planing or scaling now completed, a final polishing should be instituted, using some fine powder, such as silex, on Abbott's brushes, cuttle-fish strips for approximal root surfaces and porte-polisher, wood points and wet silex for gingival area. For a final lustre using whiting with Cutter's floss silk, porte-polisher and wood points.

Finally, brethren, let us keep this old adage ever before us: "An ounce of prevention is worth a pound of cure." Let us drop our policy of "watchful waiting" and get at this problem, each and every one determined that every preventive measure in his or her power shall be used to down this enemy. The results will be fruitful beyond all expectations, and our profession will be lifted another notch toward the high place it will some day occupy.—*Dominion Dental Journal.*

